# Severity and susceptibility: measuring the perceived effectiveness and believability of tobacco health warnings

**DOI:** 10.1186/s12889-018-5385-x

**Published:** 2018-04-10

**Authors:** Olivia M. Maynard, Harry Gove, Andrew L. Skinner, Marcus R. Munafò

**Affiliations:** 1 MRC Integrative Epidemiology Unit, University of Bristol, School of Experimental Psychology, 12a Priory Road, Bristol, BS81TU UK; 2 UK Centre for Tobacco and Alcohol Studies, School of Experimental Psychology, University of Bristol, 12a Priory Road, Bristol, BS8 1TU UK; 3 Department of Psychology, University of Bath, Bath, BA27AY UK

**Keywords:** Tobacco, Health warnings, Public policy, Packaging and labelling, Severity, Susceptibility

## Abstract

**Background:**

Pictorial tobacco health warning labels (HWLs) have been shown to be more effective than text-only HWLs in changing smoking attitudes and intentions. However, there is contradictory evidence regarding how the severity of the content of HWLs influences responses to them.

**Methods:**

We examined the perceived believability and effectiveness of HWLs in an online study using a convenience sample of non-smokers (*N* = 437) and smokers (*N* = 436). HWLs were in one of three presentation formats: (text-only, a moderately severe image or highly severe image) and focussed on three disease outcomes (lung cancer, blindness or tooth and gum disease). Participants rated the effectiveness and believability of each HWL and also rated their perceived susceptibility to each disease.

**Results:**

A 2 (smoking status) × 3 (presentation format) × 3 (disease outcome) ANOVA was run for both believability and effectiveness ratings. The most severe pictorial HWLs received the highest believability and effectiveness ratings and as expected, the text-only HWLs received the lowest. Lung cancer HWLs were rated most believable and effective, with the blindness HWLs receiving the lowest scores. A 2 (smoking status) × 3 (disease outcome) ANOVA was conducted on the ratings of perceived susceptibility to the three diseases. Smokers considered themselves to be more susceptible to all three diseases, and among smokers, perceived susceptibility to the diseases was positively correlated with effectiveness and believability ratings of the HWLs.

**Conclusion:**

Our findings support previous evidence that pictorial HWLs are rated as more effective and believable than text-only warnings, and provide some support for the use of severe or ‘grotesque’ HWLs on tobacco products. Our data also suggest that HWLs should aim to increase perceived susceptibility to disease, as this was positively related to perceived message effectiveness and believability.

**Electronic supplementary material:**

The online version of this article (10.1186/s12889-018-5385-x) contains supplementary material, which is available to authorized users.

## Background

Understanding how the features of tobacco health warning labels (HWLs) influence overall message believability and effectiveness is politically important. Indeed, the tobacco industry claimed that proposed pictorial HWLs in the USA were not simply providing ‘factual and uncontroversial information’ and instead were ‘shocking and repelling’, ultimately barring their introduction [1]. Pictorial tobacco HWLs have consistently been shown to be more effective than text-only HWLs in changing smoking attitudes and intentions [2, 3]. While graphic or ‘gruesome’ pictorial HWLs are generally found to be effective [4], there is contradictory evidence regarding how the severity of the content of HWLs and the diseases presented influences their effectiveness and believability. Previous research suggests that highly severe HWLs increase smokers’ intention to quit smoking as compared with less severe HWLs [5] and are more believable than symbolic warnings [6]. Furthermore, high emotion HWLs have been shown to elicit greater emotional reaction than lower-emotion HWLs, with emotional reaction to HWLs related to higher risk perceptions of smoking [7]. In contrast, other research on a range of different health outcomes indicates that highly severe warnings may result in defensive reactions [8–10]. This finding is supported by the Extended Parallel Process Model of fear-appeals [11] which suggests that fear appeals such as HWLs which only increase threat, without offering solutions for how to deal with this threat (i.e. efficacy messages) lead to maladaptive ‘fear control’ responses.

The degree to which a HWL is perceived as believable and effective is also likely to be influenced by the diseases presented [4, 12, 13] and one’s perceived susceptibility to developing these diseases [11, 14]. Fotuhi and colleagues found that compared to successful and failed quitters, continuing smokers had higher levels of risk-minimising beliefs [15], while Park and colleagues observed that smokers gave more uncertain answers regarding their risk of disease as compared with former smokers [16]. HWLs which include messages regarding susceptibility to smoking-related disease have also been shown to increase message effectiveness [17].

Here we aimed to understand, using a series of unfamiliar tobacco HWLs, the influence of message severity, disease outcome and perceived susceptibility to disease, on the perceived believability and effectiveness of these HWLs among smokers and non-smokers.

## Methods

### Study design and participants

In this online survey, adult smokers and non-smokers were shown nine HWLs representing three disease outcomes in three different presentation formats. Participants rated HWL effectiveness and believability and their susceptibility to the three diseases. Given the large sample size required (see the Statistical Analysis section below), the survey platform Qualtrics was used to run the survey and we recruited participants opportunistically using the online crowdsourcing platform Prolific Academic. Participants took approximately 10 min to complete the survey and were reimbursed £1 for their participation (an amount commensurate with Prolific Academic reimbursement guidelines). We pre-registered the study protocol on the Open Science Framework (https://osf.io/wuz4a/).

### Materials

#### Health warnings

We developed nine HWLs, using three disease outcomes, and three presentation formats (stimuli are available from the Bristol Research Data Repository (http://data.bris.ac.uk/data/; 10.5523/bris.381hbwlv26t9w2ipkng7f941e3). We conducted two pilot studies to determine which disease outcomes should be used (see the study protocol for more information: https://osf.io/wuz4a/). The three disease outcomes selected were: ‘Smoking causes 9 out of 10 lung cancers’, ‘Smoking increases the risk of blindness’ and ‘Smoking damages your teeth and gums’. The three presentation formats were: text-only, a ‘moderately severe’ image (i.e. an image showing moderate or average physical effects of smoking on either internal or external body parts) and a ‘highly severe’ image (i.e. an image showing a severe or worst case physical effect of smoking on either internal or external body parts). The two pilot studies were also used to determine that the images used were indeed ‘moderately’ or ‘highly’ severe, where participants were asked ‘how graphic (i.e. showing gruesome and vivid physical effects of the smoking related disease) is this picture’ and responded on a 1–10 Likert scale. Images with a mean score between 7 and 10 comprised the ‘highly severe’ images, and those with a score from 4 to below 7 comprised the moderately severe category.

#### Believability and effectiveness ratings

Perceived HWL believability was assessed using the single question ‘How believable is this health warning?’ Perceived HWL effectiveness was assessed using the single question ‘How effective is this health warning?’ To assist participants in answering this question, the following text was also provided ‘e.g., in encouraging smokers to quit, increasing concerns about smoking, and discouraging youth from starting to smoke’. These three effectiveness examples have been found to be highly correlated with questions regarding ‘overall effectiveness’ (Cronbach’s α = 0.97) [4, 6]. Believability and effectiveness questions were answered on a visual 1–10 Likert scale, with 1 labelled ‘not at all’ and 10 labelled ‘extremely’ [4, 12, 18, 19].

#### Perceived susceptibility

After viewing the HWLs, participants rated their perceived susceptibility to develop each of the three disease outcomes on a one to seven scale by answering the question ‘How likely do you think you are to suffer from each of the following diseases or ailments in the future?’ [20]. Participants reported whether they currently have this disease or have done in the past, with the following options: ‘I do not/ have not had this disease’, ‘I have had this disease in the past’ or ‘I currently have this disease’.

### Procedure

Participants provided informed consent, demographic information, smoking status and if a smoker, reported for how long they had been a smoker [21]. The nine HWLs were presented randomly and individually on screen and participants were given as long as required to view the HWL and answer the believability and effectiveness questions (the order of which was randomised). Participants then completed the perceived susceptibility questions and were provided with debriefing information and reimbursement instructions.

### Statistical analysis

A sample size calculation indicated that 872 participants would be required and details of this can be found in the study protocol. A 2 (smoking status) × 3 (presentation format) × 3 (disease outcome) ANOVA was run for both believability and effectiveness ratings. A 2 (smoking status) × 3 (disease outcome) ANOVA was conducted on the ratings of perceived susceptibility to the three diseases. For both analyses, ANOVA allowed us to examine both the main effects of our independent variables (i.e. the effects of our independent variables averaging across the levels of the other independent variables) and the interactions between them. This allows us to examine, for example, whether there are general differences in effectiveness scores between the presentation formats (i.e., the main effect of presentation format) and whether there are differences in effectiveness scores for the different presentation formats between smokers and non-smokers (i.e., the presentation format × smoking status interaction). We specified our planned use of ANOVA in our study protocol, which was published online prior to starting data collection. The data that form the basis of the results are available from the Bristol Research Data Repository, 10.5523/bris.381hbwlv26t9w2ipkng7f941e3.

## Results

### Characteristics of participants

Participants were required to be aged 18 or older, live in the UK (to ensure similar exposure to UK tobacco HWLs) and be either a non-smoker (*n* = 437) or a smoker (*n* = 436). Detailed participant characteristics are shown in Additional file 1: Table S1. Smokers and non-smokers differed on education, income and prevalence of tooth and gum disease, but not lung cancer or blindness.

### Believability and effectiveness ratings

**Fig. 1:**
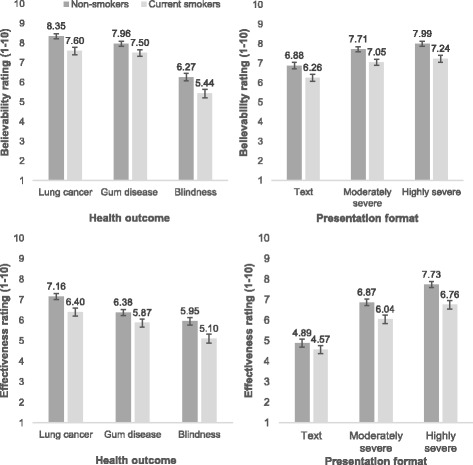
Health warning label believability and effectiveness ratings for the three disease outcomes and three presentation formats among non-smokers and smokers. Error bars represent 95% confidence intervals

Results are presented in Fig. 1. Mean believability and effectiveness scores were moderately highly correlated (*r* = 0.60, *p* < 0.001). Greenhouse-Geisser corrected statistics are reported for the main effects of outcome and presentation on our dependant variables effectiveness and believability.

Smokers gave lower believability (F_(1,871)_ = 46.74, *p* < 0.001, η_p_^2^ = 0.05) and effectiveness ratings (F_(1,871)_ = 38.51, *p* < 0.001, η_p_^2^ = 0.04) than non-smokers. A main effect of presentation format was observed for both believability (F_(2,1014)_ = 263.12, *p* < 0.001, η_p_^2^ = 2.32) and effectiveness ratings (F_(2,1348)_ = 1017, *p* < 0.001, η_p_^2^ = 0.54). Bonferroni corrected post-hoc *t*-tests indicated that highly severe pictorial HWLs were more believable (*t*_*(870)*_ = 6.37, *p* < 0.001) and effective (*t*_*(870)*_ = 18.98, *p* < 0.001) than moderately severe pictorial HWLs, which in turn were more believable (*t*_*(870)*_ = 17.08, *p* < 0.001) and effective (*t*_*(870)*_ = 30.02, *p* < 0.001) than text-only HWLs.

A main effect of disease outcome was observed for both believability (F_(2,1607)_ = 3804.0, *p* < 0.001, η_p_^2^ = 0.42) and effectiveness ratings (F_(2,1684)_ = 229.26, *p* < 0.001, η_p_^2^ = 0.21). Bonferroni corrected post-hoc *t*-tests indicated that lung cancer HWLs were more believable (*t*_*(870)*_ = 4.10, *p* < 0.001) and effective (*t*_*(870)*_ = 11.93, *p* < 0.001) than tooth and gum disease HWLs, which in turn were more believable (*t*_*(870)*_ = 138.44, *p* < 0.001) and effective (*t*_*(870)*_ = 10.30, *p* < 0.001) than blindness HWLs. Given the differences between smokers and non-smokers for income and education, we conducted an ANCOVA with income and education as covariates. This analysis allowed us to statistically control for any potential effects of these covariates on our dependent variables. This analysis did not meaningfully change the results described above. Data that comprise these analyses are available from the Bristol Research Data Repository.

### Perceived susceptibility

**Fig. 2:**
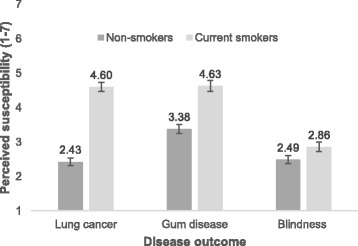
Perceived susceptibility to the three diseases among non-smokers and smokers. Error bars represent 95% confidence intervals

Greenhouse-Geisser corrected statistics for the ANOVA are reported (see Fig. 2). Data on perceived susceptibility to disease were not available for one non-smoker and five smokers. A main effect of outcome (F_(2,1709)_ = 390.70, *p* < 0.001, η_p_^2^ = 0.31) was observed, with participants rating their susceptibility to tooth and gum disease to be higher than lung cancer (*t*_*(864)*_ = 10.04, *p* < 0.001), which in turn was perceived to be higher than blindness (*t*_*(864)*_ = 18.30, *p* < 0.001). A main effect of smoking status (F_(1,865)_ = 284.09, *p* < 0.001, η_p_^2^ = 0.25) was also observed, with smokers rating their susceptibility to the diseases as higher than non-smokers.

In a series of exploratory analyses using Pearson’s correlation, we examined the relationship between perceived susceptibility to a disease and the believability and effectiveness ratings of the HWLs presenting that disease. As shown in Additional file 2: Table S2, among smokers, perceived susceptibility to each disease was positively correlated with both the believability and effectiveness scores given for the HWLs presenting that disease. However, among non-smokers, there was only evidence of a weak positive relationship between perceived susceptibility to blindness and the believability of the blindness HWLs. There was little evidence for other correlations among non-smokers.

## Discussion

We find that highly severe pictorial HWLs are perceived as being both more believable and effective than moderately severe pictorial HWLs and text-only HWLs among both smokers and non-smokers. This provides some support for the use of highly severe or ‘grotesque’ HWLs on tobacco products, as proposed by the FDA.

We find that the lung cancer HWLs were perceived as most effective and believable, while the blindness HWLs were rated least effective and believable by smokers and non-smokers. Perceived susceptibility to these diseases is likely to have influenced these ratings. Indeed, both the Extended Parallel Process Model [11] and the Risk Perception Attitude (RPA) Framework [14] indicate that perceived susceptibility to a disease is important in evoking a fear-response to a HWL. Among smokers, but not non-smokers, Pearson’s correlations indicated that perceived susceptibility to all three diseases was positively correlated with effectiveness and believability scores for the related HWL, indicating that perceived susceptibility to a disease is important in determining believability. This suggests that HWLs should not only present the most debilitating disease outcomes, but also those which smokers are most susceptible to (or those they perceive themselves to be susceptible to). HWLs could also present messages which explicitly increase individual’s perceived susceptibility to disease. Interestingly, participants rated their susceptibility to blindness as lower than both lung cancer and tooth and gum disease, reflecting their actual susceptibility [22–27] and among non-smokers, while the lung cancer and teeth and gum disease HWLs were rated as relatively highly believable and effective, the blindness HWL was only rated as believable among those who felt more susceptible to blindness. Future research should investigate which diseases smokers feel they are most susceptible to and develop HWLs which target these diseases.

Our study has a number of important strengths, in particular the large sample size including non-smokers and smokers. However, collecting data from a convenience sample such as this also represents a problem, as there is evidence that our sample had a higher level of education and income than the general population [28]. Although our ANCOVA analyses controlling for these variables did not meaningfully change our results, future research should specifically target individuals with lower income and levels of education. Our study is also limited by its reliance on self-report data and further experimental work should determine whether these self-report responses reflect participants’ actual behavioural responses to these HWLs. In addition, given that the believability and effectiveness ratings of health warnings were correlated, it is possible that our single item question for each was measuring a single underlying factor. Finally, the analysis of the relationship between susceptibility ratings and believability and effectiveness ratings was exploratory and this finding should be treated with caution until replicated, particularly as it is possible that viewing the HWLs prior to estimating perceived susceptibility to disease may have influenced these ratings. Future research should experimentally manipulate perceived susceptibility to disease to fully understand this relationship.

## Conclusions

We find that pictorial HWLs are rated as more effective and believable than text-only warnings and that highly severe pictorial warnings are rated as more effective and believable than moderately severe pictorial warnings. This supports the use of severe pictorial warnings on tobacco products, although we also note that perceived susceptibility to smoking related diseases is an important determinant in message effectiveness and believability. HWLs should therefore focus on diseases for which smokers are highly susceptible, or include messages that explicitly increase perceived susceptibility for that disease.

## Additional files

Additional file 1: **Table S1.** Characteristics of participants. (PDF 123 kb)

Additional file 2: **Table S2.** Correlations between ratings of the three health warning label outcomes and smoker and non-smoker’s perceived susceptibility to these diseases. (PDF 103 kb)

## Abbreviations

ANCOVA: Analysis of covariance
ANOVA: Analysis of variance
F: F-distribution
FDA: Food and drug administration
HWL: Health warning label
N: Number
p: Probability value (*p*-value)
r: Pearson correlation coefficient (Pearson’s r)
t: T-statistic
α: Alpha
η_p_^2^: Partial eta squared
