# Supplementary material for: Severity and susceptibility: measuring the perceived effectiveness and believability of tobacco health warnings
Source: BMC Public Health. 2018 Apr 10;18:468. doi: 10.1186/s12889-018-5385-x (PMC5894175; doi:10.1186/s12889-018-5385-x)
Supplement: Supplementary file 1 — Table S1. Characteristics of participants. (PDF 123 kb) [file 12889_2018_5385_MOESM1_ESM.pdf]

Table 1. Characteristics of participants

	Non-smokers (n = 437)	Smokers (n = 436)	P value for difference
**Gender (female)**	221 (50.6)	218 (50.0)	0.973
**Age**	32.6 (11.4)	34.1 (11.6)	0.067
**Length of time a smoker**			
'Over 5 years'		75%	
'Between 1 and 5 years'		18%	
'About a year'		6%	
'A few months or less'		1%	
**Ever prevalence of disease**			
Lung cancer	0.5%	0.9%	0.19
Blindness	0.5%	1.2%	0.11
Tooth and gum disease	8.7%	24.1%	<0.001
**Education**			<0.001
Missing	1 (0.2)	1 (0.2)	
Primary or secondary school/vocational level 1 & 2/trade apprenticeship	10 (2.3)	21 (4.8)	
Secondary school advanced/vocational level 3	38 (8.7)	66 (15.1)	
Further education/training college below degree level	71 (16.2)	106 (24.3)	
Some university	63 (14.4)	64 (14.7)	
Completed university degree	183 (41.9)	138 (31.7)	
Post-graduate degree	71 (16.2)	40 (9.2)	
**Income**			<0.001
Missing	1 (0.2)	3 (0.7)	
< £6500	20 (4.6)	19 (4.4)	
£6500 – 15,000	41 (9.4)	51 (11.7)	
£15,001 – 30,000	108 (24.7)	167 (38.3)	
£30,001 – 40,000	86 (19.7)	84 (19.3)	
£40,001 – 50,000	63 (14.4)	42 (9.6)	
£50,001 – 65,000	59 (13.5)	35 (8.0)	
£65,001 – 95,000	39 (8.9)	27 (6.2)	
> £95,000	20 (4.6)	8 (1.8)	
**Location**			0.668
Missing	1 (0.2)	4 (0.9)	
England	359 (82.2)	363 (83.3)	
Wales	26 (5.9)	29 (6.7)	
Scotland	41 (9.4)	33 (7.6)	
Northern Ireland	10 (2.3)	7 (1.6)	

For categorical variables, values represent percentage. For continuous variables, values represent mean (SD). P-values for the difference between groups was calculated using chi-square (for gender, length of time a smoker, ever prevalence of disease, education, income and location)or *t* tests (for age).
