# Supplementary material for: Severity and susceptibility: measuring the perceived effectiveness and believability of tobacco health warnings
Source: BMC Public Health. 2018 Apr 10;18:468. doi: 10.1186/s12889-018-5385-x (PMC5894175; doi:10.1186/s12889-018-5385-x)
Supplement: Supplementary file 2 — Table S2. Correlations between ratings of the three health warning label outcomes and smoker and non-smoker’s perceived susceptibility to these diseases. (PDF 103 kb) [file 12889_2018_5385_MOESM2_ESM.pdf]

Table 2. Correlations between ratings of the three health warning label outcomes and smoker and non-smoker's perceived susceptibility to these diseases.

		Non-smokers' perceived susceptibility of each disease			Smokers' perceived susceptibility of each disease		
		Blindness	Lung cancer	Tooth and gum disease	Blindness	Lung cancer	Tooth and gum disease
Believability of each HWL	Blindness	0.162<br>0.001			0.454<br><0.001		
	Lung cancer		-0.005<br>0.909			0.251<br><0.001	
	Tooth and gum disease			-0.001<br>0.980			0.370<br><0.001
Effectiveness of each HWL	Blindness	0.077<br>0.106			0.355<br><0.001		
	Lung cancer		0.096<br>0.045			0.243<br><0.001	
	Tooth and gum disease			-0.053<br>0.269			0.250<br><0.001

Values represent Pearson's correlation (r) followed by the p value for the correlation.
